# On the periodic solutions of the three-body problem

**DOI:** 10.1093/nsr/nwz102

**Published:** 2019-07-24

**Authors:** Shijun Liao, Xiaoming Li

**Affiliations:** 1 Advanced Center of Computing, School of Naval Architecture, Ocean and Civil Engineering, Shanghai Jiao Tong University, China; 2 School of Physics and Astronomy, Shanghai Jiao Tong University, China; 3 MOE Key Laboratory of Disaster Forecast and Control in Engineering, School of Mechanics and Construction Engineering, Jinan University, China

The motion of three bodies that attract each other by universal gravitation was first investigated by Newton [1] and was reconsidered by many great mathematicians and scientists, including Euler, Lagrange, Poincaré [2] and Hilbert. The famous three-body problem has had a great influence on physics, mathematics and non-linear dynamics. It has led to a new field of modern science, i.e. chaotic dynamics. Stable periodic motion of a multiple celestial body system could provide a necessary background in time and space for the evolution of living beings, because human beings might be a result of more than four billion years of evolution. So, it is important to find periodic solutions of the three-body problem.

Surprisingly, according to Montgomery's topological method [3] to classify families of three-body orbits, only three families of periodic solutions of the three-body problem were found in 300 years, i.e. (1) the Euler–Lagrange family found by Euler (1767) and Lagrange (1772); (2) the BHH family found by Broucke (1975), Hadjidemetriou (1975) and Hénon (1976); and (3) the figure-eight family numerically found by Moore (1993), until 2013, when Šuvakov and Dmitrašinović [4] found 11 families of new periodic solutions of a three-body system with equal mass by computer simulation.

Why? The mystery was revealed in 1890 by Poincaré [2], the father of chaotic dynamics, who proved the non-existence of the uniform first integral of a three-body problem in general, and more importantly, the sensitive dependence to initial conditions (SDIC) of its trajectories. Thus, Poincaré realized the importance of periodic solutions of the three-body problem: ‘what makes these periodic solutions so precious to us, is that they are, so to say, the only opening through which we can try to penetrate in a place which, up to now, was supposed to be inaccessiblersquo;.

The SDIC of a chaotic system was found again by Lorenz in the 1960s with the aid of a computer; he popularized this concept via his famous ‘butterfly effect’, i.e. a hurricane in North America might be created by the flapping of wings of a distant butterfly in South America several weeks earlier. More importantly, Lorenz also found later that the trajectory of a chaotic dynamic system has sensitive dependence not only on the initial conditions but also on numerical algorithms. Note that there are many numerical algorithms for a chaotic system, which could be quite different from each other! At each time-step, different numerical algorithms bring different truncation errors, which increase exponentially for a chaotic system due to the ‘butterfly effect’. So, for a chaotic system, the sensitive dependence on numerical algorithms is an inevitable result of the SDIC. As a result, for a chaotic system with an exact initial condition, one mostly gains quite different numerical trajectories, since numerical chaotic results given by algorithms in double precision are a kind of mixture of the ‘true’ solution and numerical noise at the same level! Such divergent simulations of chaotic systems are unacceptable for many researchers, and inescapably cause bitter controversy.

**Figure 1.**
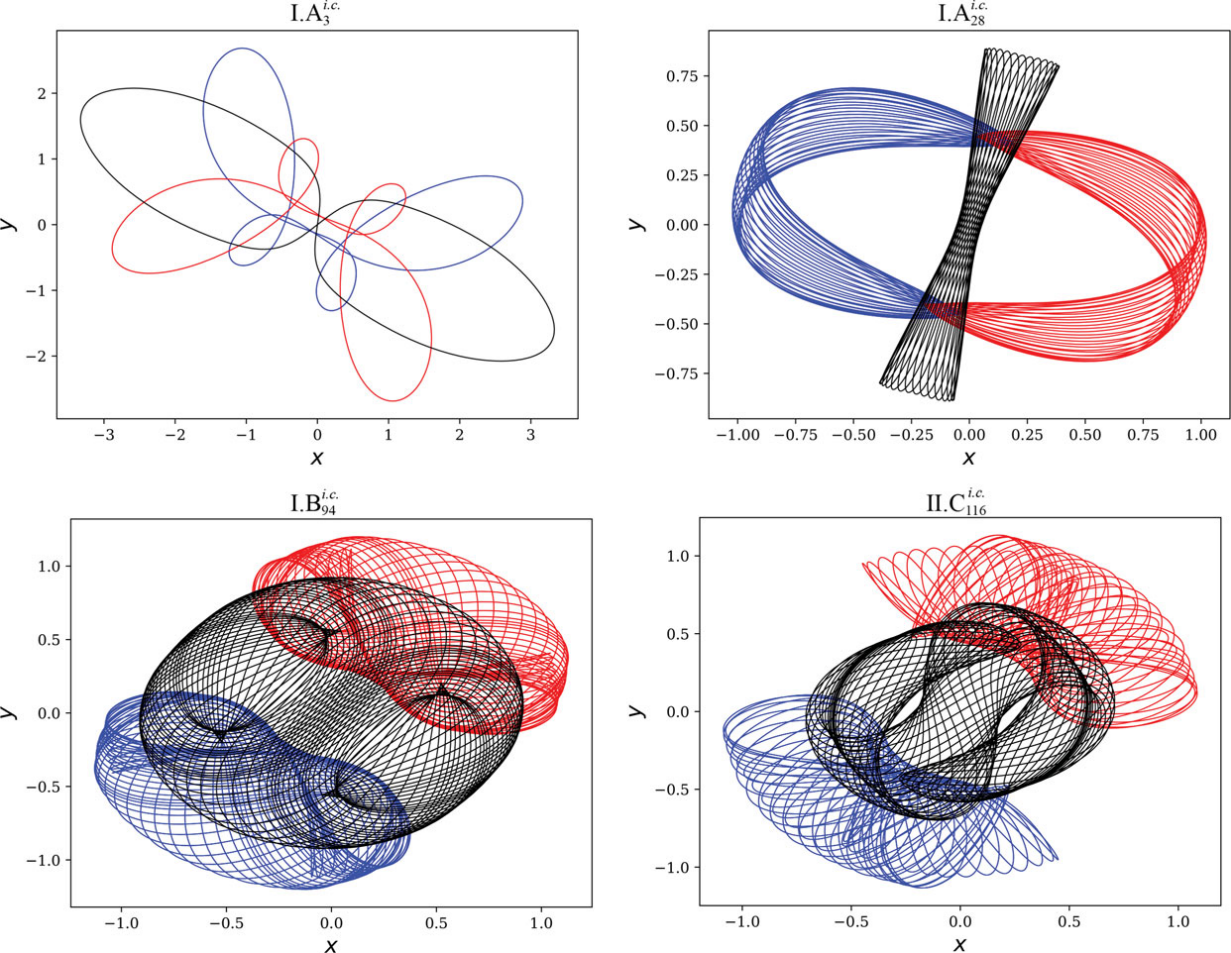
A few examples of new periodic orbits of a three-body system with equal mass. Red line: first body; black line: second body; blue line: third body. Top left: periodic orbit $I.{A}_3^{i.c.}$. Top right: periodic orbit $I.{A}_{28}^{i.c.}$. Bottom left: periodic orbit $I.{B}_{94}^{i.c.}$. Bottom right: periodic orbit $II.{C}_{116}^{i.c.}$. Movies of more than 2000 of these new periodic orbits are given on the website: http://numericaltank.sjtu.edu.cn/three-body/three-body.htm.

To overcome this obstacle, a new strategy of numerical simulation for chaotic systems was proposed by Liao [5], namely the clean numerical simulation (CNS). The CNS is based on the Taylor-series method at high enough order and data at multiple precisions with a large enough number of digits, plus a convergence check using an additional simulation with less numerical noise. In this way, both truncation and round-off errors can be reduced to an arbitrary level so that in theory convergent (reliable) chaotic solutions can be obtained in an arbitrary long (but finite) interval of time by means of the CNS. For example, the CNS can reduce numerical noise to such a tiny level, much smaller than the micro-level uncertainty of physical quantities, that propagation of these physical micro-level uncertainties of chaotic dynamic systems can be precisely investigated [6]. Thus, unlike traditional numerical algorithms, the CNS can provide convergent/reliable numerical simulations of chaotic systems in quite long intervals of time. Obviously, the CNS provides a more reliable way to gain the ‘true’ trajectory of the three-body problem in general. In 2017, using the CNS as a new tool to gain a reliable trajectory and by means of a grid search method for initial conditions and the Newton–Raphson method to modify a possible initial condition, Li and Liao [7] successfully gained 695 families of periodic solution of the three-body system with equal mass by means of a national supercomputer, including the figure-eight family found by Moore, the 11 families found by Šuvakov and Dmitrašinović, and in particular more than 600 new families that have never been reported (a few of them are shown in Figure 1). Similarly, Li *et al*. [8] found 1349 new families of periodic solution of a three-body system with only two bodies having the same mass (http://numericaltank.sjtu.edu.cn/three-body/three-body-unequal-mass.htm). Currently, Li and Liao [9] have further successfully gained 313 collisionless periodic orbits of the free-fall three-body system with some randomly chosen values of mass ratios. This strongly suggests that there should exist an infinite number of families of periodic orbits of the three-body system in general. Using the CNS, these trajectories and their corresponding periods can be at a would precision of as many digits as one like. These new periodic orbits [7–9] have been reported twice by *New Scientist* (https://www.newscientist.com/article/2170161-watch-the-weird-new-solutions-to-the-baffling-three-body-problem/).

Surprisingly, all of these periodic orbits approximately satisfy the so-called generalized Kepler's third laws [7–10]. This suggests that there should exist some elegant structures for the three-body system. Traditionally, a non-hierarchical three-body system was believed to be unstable. However, all of the new periodic orbits reported in [7–9] are in non-hierarchical configurations, but many among them are linearly or marginally stable. This might inspire the long-term astronomical observation of stable non-hierarchical triple systems in practice. All of these would enrich our knowledge and deepen our understanding about the three-body problem.

The success of the CNS with the famous three-body problem illustrates its great potential. It is quite promising that the CNS could provide a new, precise tool to investigate some challenging problems, such as energy spectra of the three-body state in quantum mechanics, turbulence, which is one of the most difficult problems in classical physics, and so on.
